# Comparison of Allergic Rhinitis Treatments on Patient Satisfaction: A MASK‐air and EAACI Methodological Committee Report

**DOI:** 10.1111/all.70055

**Published:** 2025-09-26

**Authors:** Bernardo Sousa‐Pinto, Rafael José Vieira, Antonio Bognanni, Matteo Martini, Michal Ordak, Giovanni Paoletti, Sara Gil‐Mata, Rita Amaral, Anna Bedbrook, Patrizia Bonadonna, Luisa Brussino, G. Walter Canonica, João Coutinho‐Almeida, Álvaro A. Cruz, Wienczyslawa Czarlewski, Mark Dykewicz, Mattia Giovannini, Bilun Gemicioglu, Juan Carlos Ivancevich, Ludger Klimek, Violeta Kvedariene, Desiree E. Larenas‐Linnemann, Manuel Marques‐Cruz, André Moreira, Marek Niedoszytko, Ana Margarida Pereira, Nikolaos G. Papadopoulos, Nhan Pham‐Thi, Frederico S. Regateiro, Sanna K. Toppila‐Salmi, Boleslaw Samolinski, Joaquin Sastre, Luís Taborda‐Barata, Tuuli Thomander, Ilgım Vardaloğlu Koyuncu, Arunas Valiulis, Leticia de las Vecillas, Maria Teresa Ventura, Jolanta Walusiak‐Skorupa, Yi‐Kui Xiang, Oliver Pfaar, João A. Fonseca, Torsten Zuberbier, Holger J. Schünemann, Danilo di Bona, Jean Bousquet

**Affiliations:** ^1^ MEDCIDS—Department of Community Medicine, Information and Health Decision Sciences, Faculty of Medicine University of Porto Porto Portugal; ^2^ CINTESIS@RISE—Health Research Network, Faculty of Medicine University of Porto Porto Portugal; ^3^ Department of Health Research Methods, Evidence, and Impact & Department of Medicine, Evidence in Allergy Group McMaster University Hamilton Ontario Canada; ^4^ Allergy Unit, Department of Internal Medicine University Hospital AOU delle Marche Ancona Italy; ^5^ Department of Clinical and Molecular Sciences Marche Polytechnic University Ancona Italy; ^6^ Department of Pharmacotherapy and Pharmaceutical Care, Faculty of Pharmacy Medical University of Warsaw Warsaw Poland; ^7^ Department of Biomedical Sciences Humanitas University, Pieve Emanuele Milan Italy; ^8^ Personalized Medicine, Asthma and Allergy IRCCS Humanitas Research Hospital Rozzano Italy; ^9^ Department of Cardiovascular and Respiratory Sciences, Porto Health School Polytechnic Institute of Porto Porto Portugal; ^10^ Department of Women's and Children's Health, Paediatric Research Uppsala University Uppsala Sweden; ^11^ ARIA Montpellier France; ^12^ MASK‐air Montpellier France; ^13^ Allergy Unit and Asthma Center Verona University Hospital Verona Italy; ^14^ Department of Medical Sciences University of Torino Torino Italy; ^15^ Allergy and Clinical Immunology Unit Mauriziano Hospital Torino Italy; ^16^ Fundaçao ProAR and (Faculdade de Medicina da) Universidade Federal da Bahia Salvador Bahia Brazil; ^17^ Medical Consulting Czarlewski Levallois France; ^18^ Section of Allergy and Immunology Saint Louis University School of Medicine Saint Louis Missouri USA; ^19^ Allergy Unit, Meyer Children's Hospital IRCCS Florence Italy; ^20^ Department of Health Sciences University of Florence Florence Italy; ^21^ Department of Pulmonary Diseases Istanbul University‐Cerrahpaşa, Cerrahpaşa Faculty of Medicine Istanbul Turkey; ^22^ Institute of Pulmonology and Tuberculosis Istanbul University‐Cerrahpaşa Istanbul Turkey; ^23^ Servicio de Alergia e Immunologia Clinica Santa Isabel Buenos Aires Argentina; ^24^ Department of Otolaryngology, Head and Neck Surgery Universitätsmedizin Mainz Mainz Germany; ^25^ Center for Rhinology and Allergology Wiesbaden Germany; ^26^ Institute of Clinical Medicine, Clinic of Chest Diseases and Allergology, Faculty of Medicine Vilnius University Vilnius Lithuania; ^27^ Institute of Biomedical Sciences, Department of Pathology, Faculty of Medicine Vilnius University Vilnius Lithuania; ^28^ Center of Excellence in Asthma and Allergy Médica Sur Clinical Foundation and Hospital México City Mexico; ^29^ EPIUnit‐Institute of Public Health University of Porto, and Laboratory for Integrative and Translational Research in Population Health (ITR) Porto Portugal; ^30^ Serviço de Imunoalergologia Centro Hospitalar Universitário São João Porto Portugal; ^31^ Basic and Clinical Immunology Unit, Department of Pathology, Faculty of Medicine University of Porto Porto Portugal; ^32^ Department of Allergology Medical University of Gdansk Gdansk Poland; ^33^ Allergy Unit Instituto and Hospital CUF Porto Portugal; ^34^ Allergy Department, 2nd Pediatric Clinic University of Athens Athens Greece; ^35^ Ecole Polytechnique de Palaiseau Palaiseau France; ^36^ IRBA (Institut de Recherche Bio‐Médicale des Armées) Bretigny Sur Orge France; ^37^ Université Paris Cité Paris France; ^38^ Allergy and Clinical Immunology Department, Hospitais da Universidade de Coimbra Unidade Local de Saúde de Coimbra Coimbra Portugal; ^39^ Center for Innovative Biomedicine and Biotechnology (CIBB), Faculty of Medicine University of Coimbra Coimbra Portugal; ^40^ Institute of Immunology, Faculty of Medicine University of Coimbra Coimbra Portugal; ^41^ UBIAir—Clinical & Experimental Lung Centre and CICS‐UBI Health Sciences Research Centre University of Beira Interior Covilhã Portugal; ^42^ Department of Otorhinolaryngology University of Eastern Finland and the North Savo Wellbeing Services County Kuopio Finland; ^43^ Department of Allergy, Skin and Allergy Hospital, Inflammation Center Helsinki University Hospital and University of Helsinki Helsinki Finland; ^44^ Department of Prevention of Environmental Hazards, Allergology and Immunology Medical University of Warsaw Warsaw Poland; ^45^ Allergy Service, Fundacion Jimenez Diaz Universidad Autonoma de Madrid, CIBERES‐ISCIII Madrid Spain; ^46^ RISE‐Health, Faculty of Health Sciences, and UBIAir‐Clinical & Experimental Lung Centre University of Beira Interior Covilhã Portugal; ^47^ Department of Immunoallergology Cova da Beira University Hospital Center Covilhã Portugal; ^48^ The Heart and Lung Center, Helsinki University Hospital University of Helsinki Helsinki Finland; ^49^ Faculty of Medicine University of Porto Porto Portugal; ^50^ Clinic of Asthma Allergy and Chronic Lung Diseases Vilnius Lithuania; ^51^ Institute of Clinical Medicine and Institute of Health Sciences Medical Faculty of Vilnius University Vilnius Lithuania; ^52^ Department of Allergy Hospital La Paz Institute for Health Research (IdiPAZ) Madrid Spain; ^53^ University of Bari Medical School Bari Italy; ^54^ Institute of Sciences of Food Production National Research Council (ISPA‐CNR) Bari Italy; ^55^ Department of Occupational Diseases and Environmental Health Nofer Institute of Occupational Medicine Lodz Poland; ^56^ Institute of Allergology, Charité—Universitätsmedizin Berlin Corporate Member of Freie Universität Berlin and Humboldt‐Universität Zu Berlin Berlin Germany; ^57^ Fraunhofer Institute for Translational Medicine and Pharmacology ITMP Immunology and Allergology Berlin Germany; ^58^ Shanghai Skin Disease Hospital Tongji University School of Medicine Shanghai China; ^59^ Section of Rhinology and Allergy, Department of Otorhinolaryngology, Head and Neck Surgery, University Hospital Marburg Philipps‐Universität Marburg Marburg Germany; ^60^ Michael G. DeGroote Cochrane Canada & McMaster GRADE Centres McMaster University Hamilton Ontario Canada; ^61^ Department of Medical and Surgical Science University of Foggia Foggia Italy

## Abstract

**Introduction:**

Satisfaction with treatments may affect medication adherence and use patterns, including the use of co‐medication. We aimed to compare different medications for allergic rhinitis (AR) on (i) patients' satisfaction and (ii) co‐medication use frequency.

**Methods:**

We assessed data from the mHealth app MASK‐air. We evaluated days on which users with self‐reported AR had used—alone or in co‐medication—intranasal corticosteroids (INCS), intranasal antihistamines (INAH), fixed combinations of INAH+INCS, or oral antihistamines (OAH). We built multivariable regression models to compare these different AR medication classes (as well as individual medications) on their (i) treatment satisfaction levels (measured using a specific daily visual analogue scale [‘VAS satisfaction’]) and (ii) odds of being used in co‐medication.

**Results:**

We assessed 28,177 days reported by 1691 MASK‐air users. For all medication classes, co‐medication usage was associated with lower treatment satisfaction. When used in monotherapy, OAH were associated with lower VAS satisfaction than INCS (−1.7 points; 95% CI = –2.7; –0.7) or INAH+INCS (−2.1 points; 95% CI = –3.5; –0.7). INCS displayed higher odds of being used in co‐medication than OAH (OR = 1.3; 95% CI = 1.0; 1.6) or INAH+INCS (OR = 1.3; 95% CI = 0.8; 1.8). When comparing individual intranasal medications, fluticasone furoate and fluticasone propionate tended to be more frequently used in co‐medication. Among individual OAH, desloratadine and rupatadine were associated with higher satisfaction, while fexofenadine was more frequently used in co‐medication.

**Conclusion:**

Using patient‐reported data, we evaluated different medication classes and treatments in terms of satisfaction and co‐medication frequency. These results provide key insights into the acceptability of AR treatments and will contribute to future treatment guidelines.

## Graphical abstract

This study compares different medications for allergic rhinitis on patients' satisfaction and co‐medication use frequency. mHealth data suggest that intranasal corticosteroids (INCS) and fixed combinations of intranasal antihistamines (INAH) and corticosteroids are associated with higher treatment satisfaction than oral antihistamines (OAH). Fixed combinations of INAH + corticosteroids and OAH are less frequently used in co‐medication than INCS.

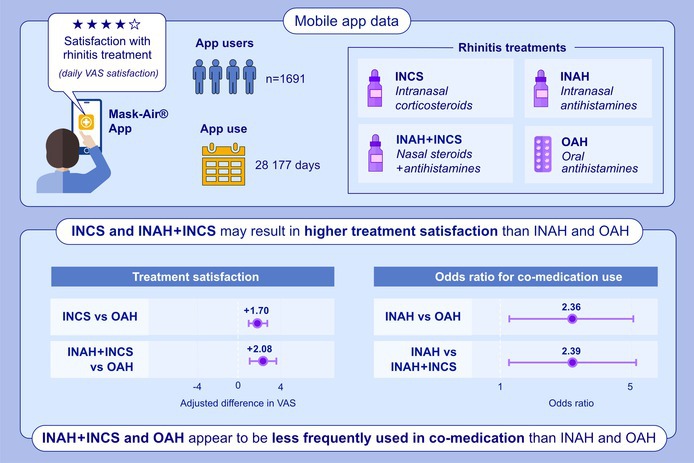

## 1 Introduction

Patients' satisfaction with their treatment (medication satisfaction) is a patient‐reported outcome measure (PROM). It is important to consider this aspect for the successful management of allergic rhinitis (AR), as it may influence treatment adherence and medication use patterns [1, 2]. In fact, if patients are not satisfied with their treatments, they may tend to switch medications or increase their doses [3, 4]. Studies assessing mobile health direct patient data have observed that AR patients frequently try different medications (even of the same class) and use co‐medication when they do not feel sufficiently well controlled [3, 4]. That is, while symptom control and treatment satisfaction are two different outcomes, they are likely to be interrelated [1].

Evaluating satisfaction is part of a comprehensive assessment of treatments that goes beyond just considering their effectiveness and safety profiles. Even though treatment satisfaction has been used as an endpoint in randomised clinical trials, few studies have compared patients' satisfaction in relation to different AR medications either directly or indirectly (by assessing variables that can be partly influenced by treatment satisfaction, such as the need for co‐medication) [5, 6]. In addition, to our knowledge, no study has ever compared different individual medications in relation to patient satisfaction using ‘real‐world’ direct patient‐generated data.

Therefore, in this study, we aimed to compare different AR medications based on their relative patients' treatment satisfaction and frequency of being used in co‐medication. In this context, we analysed MASK‐air mHealth data and compared (i) medication classes and (ii) individual agents. This study will inform the Allergic Rhinitis and its Impact on Asthma (ARIA) 2024–2025 guidelines.

## 2 Methods

### 2.1 Study Design

We conducted an observational study using direct patient data from MASK‐air. For several AR medication classes and individual medications, we compared the reported treatment satisfaction levels when such medications were used in monotherapy versus co‐medication. In addition, we compared different AR medication classes and individual medications on their (i) associated treatment satisfaction levels and (ii) odds of being used in co‐medication.

### 2.2 Setting and Participants

MASK‐air (www.mask‐air.com) has been launched in 30 countries. It is freely available on the Google Play and Apple App Stores. It is a Good Practice of the Directorate‐General for Health and Food Safety (European Commission) for digitally enabled, patient‐centred care in rhinitis and asthma multimorbidity [7, 8, 9]. It is also a Best Practice of the Organisation for Economic Cooperation and Development (OECD) for Public Health on integrated care for chronic diseases [10, 11].

We assessed MASK‐air data (i) from users aged above 16 years (or ≥ 13 years, depending on the age of digital consent in each country [12]) with self‐reported AR and (ii) reported between May 2023 (month when satisfaction to AR treatment started to be assessed in MASK‐air) and June 2024. We analysed all days on which patients (i) reported having used intranasal antihistamines (INAH), intranasal corticosteroids (INCS), fixed combinations of INAH+INCS or oral antihistamines (OAH) and (ii) had filled in the MASK‐air daily monitoring questionnaire the previous day. We applied this latter criterion to measure the level of AR control the day before medication was used. We analysed all days on which the aforementioned eligibility criteria were met.

### 2.3 Ethics

MASK‐air is *Conformité Européene* (CE) registered and follows the European Union General Data Protection Regulation. An independent review board approval was not required for this specific study because (i) the use of MASK‐air secondary data for research purposes has already been approved by an independent review board (Köln‐Bonn, Germany; reference number 17‐069), (ii) all data were anonymised before the study using k‐anonymity and (iii) users agreed to the analysis of their data for research purposes in the MASK‐air terms of use (translated into all languages and customised according to each country legislation).

### 2.4 Data Sources and Variables

MASK‐air includes a daily monitoring questionnaire in which users report (i) the impact of AR symptoms through four visual analogue scales (VASs) addressing different symptoms (0–100 scale, with higher scores corresponding to larger negative impact) (Table S1) and (ii) their AR daily medication use (available from country‐specific lists with prescribed and over‐the‐counter medications). In addition, when patients report the use of medication, they are asked how satisfied they are with their AR treatment via a VAS (‘VAS satisfaction’; 0–100 scale, with higher scores indicating higher satisfaction).

Electronic symptoms and medication data provided daily by patients allow the calculation of the allergy combined symptom‐medication score (CSMS) developed by Sousa‐Pinto et al. [13], which assesses the daily control of AR. It was found that the electronic CSMS displays a higher validity and responsiveness than the one previously defined by Pfaar et al. The CSMS is calculated based on the previously published formula: [13]

$$\begin{array}{c} {}[\left(0.037\times \mathrm{VAS}\;\text{Global Symptoms}\right)\\ +\left(0.033\times \mathrm{VAS}\;\text{Eyes}\right)+\left(0.020\times \mathrm{VAS}\;\text{Nose}\right)\\ +\left(0.027\times \mathrm{VAS}\;\text{Asthma}\right)+\left(0.450\;\text{if AzeFlu is used}\right)\\ +\left(0.424\;\text{if nasal steroids}\;\mathrm{are}\;\text{used}\right)\\ +\left(0.243\;\text{if asthma medication is used}\right)\\ +\left(0.380\;\text{if other rhinitis relief medication is used}\right)]\times 7.577 \end{array}$$

We compared VAS satisfaction levels among different medication classes and individual medications. Furthermore, we compared medications on their odds of being used in co‐medication. Accounting for patients' perspectives, we considered as days of monotherapy those when only one AR drug formulation was reported. In this context, even though they contain two active compounds, fixed combinations of INAH+INCS were considered as monotherapy (as patients use them in a single formulation). By contrast, co‐medication was considered in days on which two or more AR drug formulations—including over‐the‐counter drugs—were used.

In this study, we compared the following AR medication classes in monotherapy and co‐medication: INAH, INCS, INAH+INCS and OAH. We selected these classes, as they were the ones most frequently addressed in the final list of prioritised questions in the ARIA 2024–2025 guidelines [14]. We also assessed and compared individual medications within each class, except for INAH due to sample size limitations. Among INCS and OAH, we assessed those individual medications for which there were more than 10,000 days of use in the full MASK‐air dataset.

When responding to the MASK‐air daily monitoring questionnaire, it is not possible to skip any of the questions, and data are saved to the dataset only after the final answer. This precludes any missing data within each questionnaire.

### 2.5 Data Analysis

Categorical variables were described using absolute and relative frequencies, and continuous variables were described using medians and percentiles 25–75. All analyses were performed using software R (version 4.3.1).

We built mixed‐effects multivariable linear regression models to compare VAS satisfaction levels for each medication class and individual medication in monotherapy versus in co‐medication. For each model, we considered VAS satisfaction levels as the dependent variable, while independent variables included (i) whether medication was used in monotherapy or in co‐medication, (ii) the CSMS levels of the previous day (as a proxy variable of the AR control level before medication use) and (iii) the patient's ARIA score (range of 0–4; assesses the number of different ways in which allergy symptoms affect users [15]). These two latter variables were selected as they were identified as potentially relevant confounders, as displayed in Figure S1. In addition, we set the patient and the month of the year as random effects (i.e., we clustered observations by patient and by month of the year). We built similar mixed‐effects multivariable linear regression models comparing different medications on their VAS satisfaction levels (instead of comparing the same medication class/individual medication in monotherapy vs co‐medication). In particular, we built three models to compare (i) medication classes, (ii) individual nasal medications (i.e., individual INCS and INAH+INCS) in monotherapy and (iii) individual OAH in monotherapy.

Finally, we built mixed‐effects multivariable logistic regression models to compare the different medications' odds of being used in co‐medication. For each model, the dependent variable corresponded to whether the treatment was used in monotherapy or co‐medication, while independent variables corresponded to the medication used, the CSMS levels of the previous day, and the patient's ARIA score. The month of the year was set as a random effect. Due to lack of model convergence, we were not able to set the identification of the patient as a random effect. Therefore, to avoid analysing more than 1 day from the same patient without taking that into account, we randomly selected one observation from each patient. Again, we built three models, namely comparing (i) medication classes, (ii) individual nasal medications in monotherapy and (iii) individual OAH in monotherapy.

Multicollinearity was tested using variance inflation factors (VIF), with values ≥ 2.5 suggesting substantial multicollinearity. Goodness‐of‐fit of multivariable logistic regression models was tested using the Hosmer‐Lemeshow test, with values < 0.10 suggesting a lack of goodness‐of‐fit.

## 3 Results

We assessed 28,177 days reported by 1691 MASK‐air users (57.2% females; mean [SD] age = 42.2 [16.1] years) (Table 1; Figure S2). Most days involved the use of monotherapy (16,768; 59.5%). The most frequently used medications were OAH (17,927 days; 63.6%), followed by INCS (12,961 days; 46.0%) and INAH+INCS (6188 days; 22.0%). The distribution of days and users per country is displayed in Table S2.

**TABLE 1:** Characteristics of the assessed participants and their reported days.

Variable	Assessed sample
*N* days	28,177
*N* users (average days per user)	1691 (16.7)
Females—*N* (%)	967 (57.2)
Age—mean (range)	42.2 (17–86)
Asthma – *N* (%)	575 (34.0)
ARIA score – *N* (%)	
0	945 (55.9)
1	133 (7.9)
2	141 (8.3)
3	144 (8.5)
4	146 (8.6)
CSMS – median (P25–P75)	16.7 (8.7–27.5)
Medication use patterns—*N* days (%)	
Monotherapy	16,768 (59.5)
Co‐medication	11,409 (40.5)
Medication classes used—*N* days (%)	
INAH	380 (1.4)
INCS	12,961 (46.0)
INAH+INCS	6188 (22.0)
OAH	17,927 (63.6)
Use of asthma medication—*N* (%)	447 (26.4)

Abbreviations: ARIA, Allergic Rhinitis and its Impact on Asthma; CSMS, Combined symptom‐medication score; INAH, Intranasal antihistamines; INCS, Intranasal corticosteroids; OAH, Oral antihistamines; P25–P75, Percentiles 25–75; SD, Standard‐deviation.

Substantial multicollinearity was not detected in any of the models (all VIF < 1.04). For all models except one (comparison of individual OAH in the frequency of use in co‐medication), there was no indication that goodness‐of‐fit was observed.

### 3.1 Comparison of Treatment Satisfaction in Monotherapy Versus Co‐Medication

In monotherapy, treatment satisfaction was lower for INAH (median VAS satisfaction levels = 60; IQR = 57) than for INCS (median = 86; IQR = 26), INAH+INCS (median = 83; IQR = 24) or OAH (median = 84; IQR = 30) (Table 2). For all medication classes, after adjustment for the previous day CSMS and AR severity, co‐medication was associated with decreased treatment satisfaction. This difference was highest for INAH (−8.8; 95% CI = –14.6; –3.08; *p* value = 0.003), followed by INCS (−2.3; 95% CI = –3.19; –1.39; *p* value < 0.001), INAH+INCS (−2.3; 95% CI = –3.5; –1.1; *p* value < 0.001) and OAH (−1.3; 95% CI = –2.0; –0.5; *p* = 0.001). For most individual medications, co‐medication was also associated with a decrease in treatment satisfaction, with the largest differences having been observed for levocetirizine, desloratadine and fluticasone furoate (Table 2).

**TABLE 2:** Levels of the visual analogue scale (VAS) on treatment satisfaction for each medication class and individual medication in monotherapy versus co‐medication.

Medication	Monotherapy—median (P25–P75) [*n*]	Co‐medication—median (P25–P75) [*n*]	Monotherapy versus co‐medication adjusted coefficient (95% CI) [*p* value]
INAH	60 (25–82) [81]	80 (56–94) [299]	−8.82 (−14.56; –3.08) [0.003]
INCS	86 (67–93) [6174]	83 (65–02) [6787]	−2.29 (−3.19; –1.39) [< 0.001]
Budesonide	84 (66–93) [316]	84 (65–89) [898]	−1.36 (−4.85; 2.13) [0.446]
Fluticasone Furoate	77 (65–90) [1194]	81 (68–91) [1195]	−3.12 (−5.69; –0.55) [0.017]
Fluticasone Propionate	90 (75–90) [318]	87 (77–97) [716]	0.00 (−2.88; 2.88) [0.999]
Mometasone Furoate	88 (70–94) [4072]	82 (62–92) [3710]	−2.13 (−3.23; –1.03) [< 0.001]
INAH+INCS	83 (67–91) [2834]	71 (19–90) [3354]	−2.27 (−3.49; –1.05) [< 0.001]
Azelastine‐Fluticasone	82 (67–91) [2694]	72 (19–90) [3208]	−2.37 (−3.62; –1.12) [< 0.001]
Olopatadine‐Mometasone	90 (77–95) [140]	55 (21–76) [146]	−2.32 (−7.77;3.13) [0.404]
OAH	84 (63–93) [7679]	80 (55–92) [10248]	−1.26 (−1.99; –0.53) [0.001]
Bilastine	85 (55–92) [1745]	82 (62–89) [2090]	−0.42 (−1.75; 0.91) [0.537]
Cetirizine	90 (67–96) [876]	72 (50–93) [1048]	−0.57 (−3.08; 1.94) [0.654]
Desloratadine	82 (66–91) [1816]	79 (56–93) [2359]	−3.75 (−5.26; –2.24) [< 0.001]
Ebastine	77 (49–92) [630]	85 (70–93) [1194]	−0.05 (−2.03; 1.93) [0.964]
Fexofenadine	95 (89–97) [317]	85 (73–94) [655]	0.87 (−2.05; 3.79) [0.560]
Levocetirizine	87 (72–92) [652]	90 (66–95) [721]	−5.03 (−7.85; –2.21) [< 0.001]
Loratadine	77 (54–92) [295]	75 (52–90) [414]	−4.56 (−9.24; 0.12) [0.057]
Rupatadine	82 (65–90) [1131]	64 (13–88) [1784]	0.15 (−2.50; 2.80) [0.912]

Abbreviations: CI, Confidence intervals; INAH, Intranasal antihistamines; INCS, Inntranasal corticosteroids; INAH+INCS, Intranasal antihistamines and intranasal corticosteroids fixed combination; OAH, Oral antihistamines; P25–P75, Percentiles 25–75.

### 3.2 Comparison of Treatment Satisfaction Among Medication Classes

In adjusted analyses, and when used in monotherapy, INCS and INAH+INCS were associated with increased satisfaction compared to INAH and OAH. In particular, INCS showed an average of 2.1 more points (95% CI = –2.9; 7.0; *p* value = 0.409) in VAS satisfaction levels compared to INAH, and of 1.7 more points (95% CI = 0.7; 2.7; *p* value = 0.001) compared to OAH (Table 3). On the other hand, INAH+INCS were associated with an average of 2.5 more points (95% CI = –2.6; 7.5; *p* value = 0.340) in VAS satisfaction levels compared to INAH, and of 2.1 more points (95% CI = 0.7; 3.5; *p* value = 0.003) compared to OAH. INCS, INAH+INCS and OAH were associated with increased satisfaction levels compared to INCS+OAH.

**TABLE 3:** Results of multivariable regression models comparing different treatment classes on (A) VAS satisfaction levels and (B) odds of each treatment being used in co‐medication. Results are displayed as differences or odds ratios for the interventions in each column versus those in each row.^a^

A. Adjusted difference in VAS levels (95% confidence intervals) [*p* value]				
**INAH**	—	—	—	—
−2.07 (−6.99;2.85) [0.409]	**INCS**	—	—	—
−2.45 (−7.49;2.59) [0.340]	−0.39 (−1.86;1.08) [0.610]	**INAH + INCS**	—	—
−0.37 (−5.29;4.55) [0.883]	**1.70 (0.68;2.72) [0.001]**	**2.08 (0.71;3.45) [0.003]**	**OAH**	—
1.36 (−3.58;6.30) [0.590]	**3.43 (2.47;4.39) [< 0.001]**	**3.81 (2.34;5.28) [< 0.001]**	**1.73 (0.89;2.57) [< 0.001]**	**INCS + OAH**
B. Adjusted odds ratio of each treatment class being used in co‐medication (95% confidence intervals) [*p* value]				
**INAH**	—	—	—	NA
1.88 (0.87;4.03) [0.109]	**INCS**	—	—	NA
**2.39 (1.07;5.33) [0.031]**	1.27 (0.91;1.77) [0.148]	**INAH + INCS**	—	NA
**2.36 (1.10;5.08) [0.028]**	1.26 (0.99;1.59) [0.067]	0.98 (0.72;1.34) [0.912]	**OAH**	NA
NA	NA	NA	NA	**INCS + OAH**

Abbreviations: INAH, Intranasal antihistamines; INCS, Intranasal corticosteroids; INAH+INCS, Fixed combination of intranasal antihistamines and corticosteroids; NA, Not applicable (since INCS+OAH already corresponds to co‐medication); OAH, Oral antihistamines; VAS, Visual analogue scale. Shaded cells indicate names of medication classes and bold values indicate significant values at a level of 0.05.

^a^ In Table 3A, the content of each cell should be interpreted as the difference in VAS satisfaction levels when comparing the treatment class in the respective column with the treatment class in the respective row. A negative value indicates that the treatment class in the column is associated with lower VAS satisfaction than the treatment class in the row. A positive value indicates higher VAS satisfaction associated with the treatment class in the columns. For example, the average VAS satisfaction level with INAH is 2.07 points below that observed with INCS. On the other hand, INAH is associated with an average VAS satisfaction that is 1.36 points higher than that for INCS+OAH. In Table 3B, the content of each cell should be interpreted as the odds ratio of co‐medication use when comparing the treatment class in the respective column with the treatment class in the respective row. A value below 1 indicates that the treatment class in the column is associated with lower odds of use in co‐medication than the treatment class in the row. A value above 1 indicates higher odds of use in co‐medication associated with the treatment class in the columns. For example, INAH is associated with 1.88 higher odds of being used in co‐medication compared to INCS.

When comparing individual intranasal medications, differences in treatment satisfaction tended to be limited (Table 4). Minor differences were also observed when comparing different individual OAHs, except for the comparisons involving desloratadine or rupatadine, which were associated with the highest VAS satisfaction levels (Table 5).

**TABLE 4:** Results of multivariable regression models comparing intranasal medications on (A) visual analogue scale (VAS) satisfaction levels and (B) odds of each treatment being used in co‐medication. Results are displayed as differences or odds ratios for the interventions in each column versus those in each row.^a^

A. Adjusted difference in VAS levels (95% confidence interval) [*p* value]					
**Azelastine‐fluticasone**	—	—	—	—	—
−0.80 (−4.19; 2.59) [0.644]	**Budesonide**	—	—	—	—
0.36 (−2.27; 2.99) [0.786]	1.16 (−2.78; 5.10) [0.563]	**Fluticasone furoate**	—	—	—
0.19 (−6.42; 6.80) [0.955]	0.99 (−6.22; 8.20) [0.787]	−0.17 (−6.99; 6.65) [0.961]	**Fluticasone propionate**	—	—
1.73 (−0.58; 4.04) [0.142]	2.53 (−1.04; 6.10) [0.163]	1.37 (−1.14; 3.88) [0.285]	1.54 (−5.08; 8.16) [0.649]	**Mometasone**	—
2.47 (−2.18; 7.12) [0.298]	3.27 (−1.88; 8.42) [0.213]	2.11 (−2.83; 7.05) [0.403]	2.28 (−5.56; 10.12) [0.569]	0.74 (−3.92; 5.40) [0.755]	**Olopatadine‐mometasone**
**B. Adjusted odds ratio of each treatment being used in co‐medication**					
**Azelastine‐fluticasone**	—	—	—	—	—
1.36 (0.76, 2.45) [0.299]	**Budesonide**	—	—	—	—
**0.63 (0.42, 0.95) [0.027]**	**0.46 (0.24, 0.88) [0.019]**	**Fluticasone furoate**	—	—	—
0.57 (0.29, 1.11) [0.098]	**0.42 (0.18, 0.95) [0.040]**	0.92 (0.46, 1.87) [0.808]	**Fluticasone propionate**	—	—
0.98 (0.72, 1.34) [0.885]	0.71 (0.40, 1.28) [0.261]	**1.57 (1.04, 2.37) [0.032]**	1.70 (0.89, 3.24) [0.110]	**Mometasone**	—
1.31 (0.56, 3.04) [0.526]	0.96 (0.36, 2.56) [0.938]	2.10 (0.87, 5.06) [0.103]	2.29 (0.83, 6.35) [0.113]	1.35 (0.58, 3.14) [0.490]	**Olopatadine‐mometasone**

^a^ In Table 4A, the content of each cell should be interpreted as the difference in VAS satisfaction levels when comparing the treatment in the respective column with the treatment in the respective row. A negative value indicates that the treatment in the column is associated with lower VAS satisfaction than the treatment in the row. A positive value indicates higher VAS satisfaction associated with the treatment in the columns. For example, the average VAS satisfaction level with azelastine‐fluticasone is 0.80 points below that observed with budesonide. On the other hand, azelastine‐fluticasone is associated with an average VAS satisfaction which is 2.47 points higher than that for olopatadine‐mometasone. In Table 4B, the content of each cell should be interpreted as the odds ratio of co‐medication use when comparing the treatment in the respective column with the treatment in the respective row. A value below 1 indicates that the treatment in the column is associated with lower odds of use in co‐medication than the treatment in the row. A value above 1 indicates higher odds of use in co‐medication associated with the treatment in the columns. For example, azelastine‐fluticasone is associated with 1.36 higher odds of being used in co‐medication compared to budesonide. Shaded cells indicate names of individual medications and bold values indicate significant values at a level of 0.05.

**TABLE 5:** Results of multivariable regression models comparing oral antihistamines on (A) visual analogue scale (VAS) satisfaction levels and (B) odds of each treatment being used in co‐medication. Results are displayed as differences or odds ratios for the interventions in each column versus those in each row.^a^

A. Adjusted difference in VAS levels (95% confidence interval) [*p* value]							
**Bilastine**	—	—	—	—	—	—	—
−1.71 (−5.51; 2.09) [0.377]	**Cetirizine**	—	—	—	—	—	—
**−3.80 (−7.15; –0.45) [0.027]**	−2.09 (−5.66; 1.48) [0.250]	**Desloratadine**	—	—	—	—	—
0.19 (−4.30; 4.68) [0.934]	1.90 (−2.90; 6.70) [0.439]	3.99 (−0.42; 8.40) [0.076]	**Ebastine**	—	—	—	—
−3.1 (−9.55; 3.35) [0.346]	−1.39 (−8.07; 5.29) [0.683]	0.69 (−5.60; 6.98) [0.829]	−3.29 (−10.42; 3.84) [0.366]	**Fexofenadine**	—	—	—
0.33 (−3.55; 4.21) [0.868]	2.04 (−1.94; 6.02) [0.314]	**4.13 (1.11; 7.15) [0.008]**	0.14 (−4.68; 4.96) [0.955]	3.43 (−3.18; 10.04) [0.309]	**Levocetirizine**	—	—
0.21 (−3.61; 4.03) [0.915]	1.92 (−1.90; 5.74) [0.325]	**4.00 (0.67;7.33) [0.019]**	0.02 (−4.66; 4.70) [0.994]	3.31 (−3.33; 9.95) [0.328]	−0.12 (−3.65; 3.41) [0.947]	**Loratadine**	—
−3.15 (−6.99; 0.69) [0.109]	−1.44 (−5.71; 2.83) [0.508]	0.65 (−3.25; 4.55) [0.745]	−3.34 (−8.32; 1.64) [0.189]	−0.05 (−6.81; 6.71) [0.989]	−3.48 (−7.81; 0.85) [0.116]	−3.36 (−7.52; 0.80) [0.114]	**Rupatadine**
B. Adjusted odds ratio of each treatment being used in co‐medication (95% confidence interval) [*p* value]							
**Bilastine**	—	—	—	—	—	—	—
1.16 (0.79, 1.72) [0.436]	**Cetirizine**	—	—	—	—	—	—
1.17 (0.82, 1.67) [0.360]	1.01 (0.68, 1.49) [0.973]	**Desloratadine**	—	—	—	—	—
0.76 (0.48, 1.19) [0.224]	0.64 (0.39, 1.05) [0.079]	0.64 (0.41, 1.01) [0.055]	**Ebastine**	—	—	—	—
0.58 (0.31, 1.09) [0.091]	**0.50 (0.26, 0.95) [0.036]**	**0.50 (0.27, 0.93) [0.028]**	0.77 (0.39, 1.53) [0.463]	**Fexofenadine**	—	—	—
0.84 (0.52, 1.34) [0.439]	0.71 (0.44, 1.16) [0.181]	0.71 (0.45, 1.12) [0.143]	1.11 (0.64, 1.91) [0.722]	1.43 (0.71, 2.90) [0.313]	**Levocetirizine**	—	—
1.45 (0.84, 2.51) [0.190]	1.23 (0.70, 2.18) [0.470]	1.23 (0.71, 2.14) [0.462]	**1.92 (1.02, 3.59) [0.041]**	**2.48 (1.16, 5.34) [0.019]**	1.73 (0.93, 3.25) [0.086]	**Loratadine**	—
1.07 (0.71, 1.62) [0.742]	0.91 (0.59, 1.41) [0.700]	0.91 (0.61, 1.38) [0.649]	1.42 (0.87, 2.32) [0.170]	1.84 (0.95, 3.58) [0.070]	1.28 (0.77, 2.14) [0.332]	0.74 (0.41, 1.33) [0.318]	**Rupatadine**

^a^ In Table 5A, the content of each cell should be interpreted as the difference in VAS satisfaction levels when comparing the treatment in the respective column with the treatment in the respective row. A negative value indicates that the treatment in the column is associated with lower VAS satisfaction than the treatment in the row. A positive value indicates higher VAS satisfaction associated with the treatment in the columns. For example, the average VAS satisfaction level with bilastine is 1.71 points below that observed with cetirizine. On the other hand, bilastine is associated with an average VAS satisfaction that is 0.19 points higher than that for ebastine. In Table 5B, the content of each cell should be interpreted as the odds ratio of co‐medication use when comparing the treatment in the respective column with the treatment in the respective row. A value below 1 indicates that the treatment in the column is associated with lower odds of use in co‐medication than the treatment in the row. A value above 1 indicates higher odds of use in co‐medication associated with the treatment in the columns. For example, bilastine is associated with 1.16 higher odds of being used in co‐medication compared to cetirizine. Shaded cells indicate the names of individual medications and bold values indicate significant values at a level of 0.05.

### 3.3 Comparison of Medication Classes on Their Chances of Being Used in Co‐Medication

In adjusted analyses, INAH were associated with higher odds of being used in co‐medication when compared to INCS (OR = 1.9; 95% CI = 0.9; 4.0; *p* value = 0.109), INAH+INCS (OR = 2.4; 95% CI = 1.1; 5.3; *p* value = 0.031) and OAH (OR = 2.4; 95% CI = 1.1; 5.1; *p* value = 0.028) (Table 3). In addition, INCS also tended to be more frequently used in co‐medication than INAH+INCS (OR = 1.3; 95% CI = 0.9; 1.8; *p* value = 0.148) or OAH (OR = 1.3; 95% CI = 1.0; 1.6; *p* value = 0.067).

When comparing individual intranasal medications, fluticasone furoate and fluticasone propionate tended to be associated with increased odds of being used in co‐medication, particularly when compared to azelastine‐fluticasone, budesonide, mometasone and olopatadine‐mometasone (Table 4). Among individual OAH, fexofenadine was the medication most frequently associated with increased odds of being used in co‐medication (Table 5).

## 4 Discussion

In this study, we used daily patient‐generated data to compare different AR medications on treatment satisfaction and on their frequency of use in co‐medication. We obtained results adjusted for the CSMS of the previous day and the baseline severity of AR, in order to account for relevant confounders. Overall, these study findings showed that: (i) co‐medication was associated with lower treatment satisfaction, (ii) INCS and INAH+INCS may result in higher treatment satisfaction than INAH or OAH and (iii) INAH+INCS and OAH were less frequently used in co‐medication than INAH or INCS. Moreover, we were able to evaluate the differences between medications.

This study complements a previous one, showing a weak correlation between VAS satisfaction and VAS on nasal or ocular symptoms, suggesting that treatment satisfaction is a different PROM to symptom control [16]. While our previous study had compared median VAS satisfaction levels in monotherapy and co‐medication, only non‐adjusted differences were assessed. In this study, we built multivariable models to compute adjusted differences—so as to better account for presumed confounding—and compared medication classes and individual medication on their use in co‐medication.

For all medication classes, co‐medication was associated with lower treatment satisfaction than monotherapy. It is unlikely that these results can be explained by co‐medication being less effective than monotherapy. It is more likely that patients use co‐medication because of low satisfaction with monotherapy. This is in line with previous MASK‐air studies showing that co‐medication days were associated with worse AR symptoms than days under monotherapy or no medication [3, 4], as well as with previous observational studies that observed worse outcomes with co‐medication strategies, independently of the severity of symptoms before treatment [17]. Of note, this study does not allow the assessment of the causal effect of satisfaction on the use of co‐medication (as this would require knowledge of AR control immediately after taking the first medication). Alternative explanations, such as the increased medication burden or the higher risk of side effects with co‐medication resulting in lower satisfaction, cannot be excluded.

We observed that treatment satisfaction was higher for INCS and INAH+INCS than for INAH or OAH. This pattern is similar to that observed when comparing these different medication classes in terms of their effectiveness. In fact, a set of systematic reviews of randomised controlled trials conducted by our team to inform the ARIA guidelines has concluded that (i) intranasal treatments (particularly INCS) are more effective than OAH in improving nasal symptoms and the rhinoconjunctivitis‐related quality of life [18] and (ii) INAH+INCS and INCS are more effective than INAH [19, 20]. These results point to the interrelation between satisfaction and medication effectiveness, even though these represent two distinct dimensions. In fact, a systematic review on patients' values and preferences in AR has found that patients place a higher value on clinical improvement rather than on potential side effects from the medications [21]. Of note, when comparing individual medications of the same class, estimates tended to be less precise (reflecting sample size limitations), and differences in treatment satisfaction were mostly minor (although a minimal important difference for VAS satisfaction to contextualise its differences has not yet been determined). We also observed that INAH+INCS and OAH were less frequently used in co‐medication than INCS and INAH. Overall, and considering that the use of co‐medication may reflect (at least in part) treatment satisfaction, these results suggest that—among all compared classes—INAH+INCS is the class with the highest acceptability to people with AR, while INAH is the class with the lowest acceptability. However, there are other aspects that should be considered (e.g., patients' preferences on the route of administration and physicians' acceptability in prescribing medications of the different classes) for further investigation. In addition, treatment satisfaction may influence not only the use of co‐medication but also medication adherence and switching. However, assessment of these aspects requires longitudinal studies.

For the first time, we compared different medication classes or individual medications on treatment satisfaction as well as on the chances of being used in co‐medication while accounting for relevant confounders. In particular, we hypothesised that the two most important factors determining AR medication use patterns are (i) baseline disease severity (as patients with more severe disease may more often seek medical attention for their AR, most probably being treated with more effective medications) and (ii) periodic fluctuations of disease symptoms (patients tend to use medication more often when feeling less well controlled) (Figure S1). In an observational context, comparing different treatments without accounting for these two factors would result in highly biased estimates. Results would more likely reflect the differences in the conditions leading to different medication use rather than the actual differences in effect across medications (Table 6 shows the imbalance in medication use patterns [exposure], satisfaction and co‐medication [outcomes] according to the categories of confounding variables). In our models, we were able to account for these two factors, even though some residual confounding may exist, as (i) the ARIA score may not fully capture disease severity and (ii) the previous day CSMS may not be a perfect indicator of pre‐medication symptoms. We were able to evaluate the distribution of other demographic and clinical variables per treatment class and in relation to VAS satisfaction levels. Such variables do not seem to account for relevant confounding, as they are not consistently imbalanced in relation to exposure and outcome (Table S3). In addition, in our models, we clustered observations by patient and month of the year, taking into account the inter‐individual and intra‐individual variability of AR.

**TABLE 6:** Treatment satisfaction and frequency of days using co‐medication and medications for each class according to (i) CSMS levels of the previous day and (ii) categories of the ARIA score.

A. CSMS levels of the previous day			
Variable	Good control—CSMS < 15.8 (*N* days = 14,237)	Medium control—15.8 ≤ CSMS ≤ 35.3 (*N* days = 9109)	Poor control—CSMS > 35.3 (*N* days = 4831)
VAS satisfaction levels—median (P25–P75)	91 (85–96)	76 (62–84)	44 (17–58)
Days using co‐medication—*N* (%)	4747 (33.3)	4051 (44.5)	2611 (54.0)
Days using medication from each class—*N* (%)			
INAH	179 (1.3)	102 (1.1)	99 (2.0)
INCS	6554 (46.0)	4662 (51.2)	1745 (36.1)
INAH+INCS	2429 (17.1)	2059 (22.6)	1700 (35.2)
OAH	8905 (62.5)	5533 (60.7)	3489 (72.2)

B. ARIA score			
Variable	ARIA score 0 (*N* days = 19,373)	ARIA score 1–2 (*N* days = 7691)	ARIA score 3–4 (*N* days = 8070)
VAS satisfaction levels—median (P25–P75)	83 (61–93)	86 (72–93)	74 (53–88)
Days using co‐medication—*N* (%)	5615 (29.0)	2572 (33.4)	2599 (32.2)
Days using medication from each class—*N* (%)			
INAH	125 (0.9)	87 (1.1)	133 (1.6)
INCS	6109 (42.9)	3006 (39.1)	2731 (33.8)
INAH+INCS	3880 (27.3)	930 (12.1)	1211 (15.0)
OAH	9259 (65.0)	3668 (47.7)	3995 (49.5)

Abbreviations: ARIA, Allergic Rhinitis and its Impact on Asthma; CSMS, Combined symptom‐medication score; INAH, Intranasal antihistamines; INCS, Intranasal corticosteroids; OAH, Oral antihistamines; P25–P75, Percentiles 25–75; VAS, Visual analogue scale.

From a methodological point of view, this study is an example of how evidence from direct patient‐generated data can be used to inform guideline recommendations. Our results will support panel members of the Allergic Rhinitis and its Impact on Asthma (ARIA) 2024–2025 guidelines in making judgements on the acceptability of AR interventions. Other decision‐making criteria ‐ for which evidence from MASK‐air data has been used ‐ include ‘values’ and ‘resources required’, as MASK‐air has allowed for estimating utility values and indirect costs associated with different levels of AR control [21, 22, 23]. Of note, the use of the so‐called ‘real‐world’ data to inform the different criteria of the Evidence‐to‐Decision framework has been the subject of active discussion by the GRADE working group, with the ARIA 2024–2025 guidelines being presented as an example in that context (Bognanni et al., in preparation).

This study has some limitations. First, we were not able to obtain information on the AR control levels immediately before medication use, something which appears to be a key determinant of medication use patterns. In fact, previous studies have suggested that frequently patients do not use medication on a daily basis, but rather when they feel less well controlled [3, 4]. As a proxy variable for AR control immediately before medication use, we considered the CSMS levels registered on the day before. While the CSMS of the previous day is expected to be related to the pre‐medication symptoms, it is important to highlight that the intensity of AR symptoms can widely vary on a day‐to‐day basis (even within the same individual), depending on many factors, including exposure to pollens or pollutants [24]. Another limitation is that the sample size was smaller than for previous MASK‐air studies, due to the recent implementation in MASK‐air of the VAS assessing satisfaction. Implications of this fact include (i) the relatively limited number of observations on INAH use (or on the use of some individual medications), which may have precluded certain associations of being considered statistically significant according to traditionally used criteria and (ii) convergence problems in the models built for estimating the odds of co‐medication (leading to the use of only one observation per patient, instead of all observations). Of note, this number of participants was also discrepant when considering different countries. This may have implications, as the availability, prescription patterns, costs and reimbursement of medications vary across countries. Finally, even though there are no missing data within each daily monitoring questionnaire, patients may preferentially tend to report data in MASK‐air when feeling less well controlled. However, this selection bias in terms of reported days is not expected to differentially affect the medications being compared.

This study also has important strengths. First, we were able to build multivariable models to better account for confounding and to more adequately compare medications both on a class level and individually. Second, the CSMS has demonstrated high validity, reliability and accuracy [13]. While the VAS on treatment satisfaction has not been assessed in its properties, the other VASs of the MASK‐air daily monitoring questionnaire have demonstrated high validity and reliability [25]. Finally, this study provides both novel and relevant information, as it compared different medication classes and individual medications on outcomes which are relevant from a patient perspective (complementing those classically used to assess medication efficacy and safety). This is in line with the ARIA 2024–2025 guidelines, which will be innovative by (i) adopting a patient‐centred perspective [14, 26] and (ii) providing recommendations on individual therapeutic agents as well as medication classes [14, 19, 20].

In conclusion, in this study, we compared different AR medications on patient‐reported satisfaction and on their frequency of use in co‐medication. We performed analyses comparing not only different treatment classes, but also individual medications. Overall, we observed that INCS and INAH+INCS may result in higher treatment satisfaction than INAH and OAH. On the other hand, INAH+INCS and OAH appear to be less frequently used in co‐medication than other treatment classes. While this study has some inherent limitations, its results can inform judgements of acceptability in the ARIA 2024–2025 guidelines, contributing to the incorporation of patients' perspectives in the formulation of recommendations, alongside other criteria such as those related to the efficacy, safety, availability and affordability of the interventions.

## Author Contributions

Bernardo Sousa‐Pinto and Rafael José Vieira participated in the study design, methodology, data analysis and manuscript writing. Antonio Bognanni, Matteo Martini, Michal Ordak, Giovanni Paoletti, Sara Gil‐Mata, Holger J. Schünemann and Danilo di Bona participated in the methodology and manuscript review. Jean Bousquet participated in the study design, methodology and manuscript review. All other authors participated in the inclusion of participants and manuscript review.

## Conflicts of Interest

J. Bousquet reports personal fees from Cipla, Menarini, Mylan, Novartis, Purina, Sanofi‐Aventis, Teva, Noucor, other from KYomed‐Innov, other from MASK‐air SAS, outside the submitted work.

A. Cruz reports personal fees from AstraZeneca, personal fees from Boehringer Ingelheim, personal fees from Chiesi, personal fees from Farmoquímica, personal fees from Eurofarma, personal fees from Aché, personal fees from GSK, personal fees from Sanofi, outside the submitted work.

G. Paoletti reports other from LoFarma, GSK, Astrazeneca, outside the submitted work.

J. C. Ivancevich reports personal fees from Laboratorios Casasco Argentina, outside the submitted work.

N. Papadopoulos reports grants from Capricare, Nestle, Numil, Vianex, REG, other from Abbott, Abbvie, Astra Zeneca, GSK, HAL, Medscape, Menarini/Faes Farma, Mylan, Novartis, Nutricia, OM Pharma, Regeneron/Sanofi, outside the submitted work.

O. Pfaar reports grants and personal fees from ALK‐Abelló, grants and personal fees from Allergopharma, grants and personal fees from Stallergenes Greer, grants and personal fees from HAL Allergy Holding B.V./HAL Allergie GmbH, grants and personal fees from Bencard Allergie GmbH/Allergy Therapeutics, grants and personal fees from Laboratorios LETI/LETI Pharma, grants and personal fees from GlaxoSmithKline, personal fees from ROXALL Medizin, personal fees from Novartis, grants and personal fees from Sanofi‐Aventis and Sanofi‐Genzyme, personal fees from Med Update Europe GmbH, personal fees from streamedup! GmbH, grants and personal fees from Pohl‐Boskamp, grants from Inmunotek S.L., personal fees from John Wiley and Sons, AS, personal fees from Paul‐Martini‐Stiftung, personal fees from RG Aerztefortbildung, personal fees from Institut für Disease Management, personal fees from Springer Publisher, grants and personal fees from AstraZeneca, personal fees from IQVIA Commercial, personal fees from Ingress Health, personal fees from Wort&Bild Verlag, personal fees from Verlag ME, personal fees from Procter&Gamble, personal fees from ALTAMIRA, personal fees from Meinhardt Congress GmbH, personal fees from Deutsche Forschungsgemeinschaft, personal fees from Thieme Publisher, grants from Deutsche AllergieLiga e.V., personal fees from AeDA, personal fees from Alfried‐Krupp Krankenhaus, personal fees from Red Maple Trials Inc., personal fees from Königlich Dänisches Generalkonsulat, personal fees from Medizinische Hochschule Hannover, personal fees from ECM Expro&Conference Management, personal fees from Technical University Dresden, grants and personal fees from Lilly, personal fees from Japanese Society of Allergy, personal fees from Forum für Medizinische Fortbildung, personal fees from Dustri‐Verlag, personal fees from Pneumolive, grants and personal fees from ASIT Biotech, grants and personal fees from LOFARMA, personal fees from Paul‐Ehrlich‐Institut, personal fees from Almirall, personal fees from Blueprint, personal fees from Cliantha, outside the submitted work; and Vice President and member of EAACI Excom, member of ext. board of directors DGAKI; coordinator, main‐ or co‐author of different position papers and guidelines in rhinology, allergology and allergen immunotherapy; associate editor (AE) of Allergy and Editor‐in‐Chief (EIC) of Clinical Translational Allergy.

J. Sastre reports grants and personal fees from SANOFI, personal fees from GSK, personal fees from NOVARTIS, personal fees from ASTRA ZENECA, personal fees from MUNDIPHARMA, personal fees from FAES FARMA, outside the submitted work.

T. Thomander reports grants from The Finnish ORL‐HNS Foundation, grants from The Research Foundation of the Pulmonary Diseases, grants from The Foundation of the Finnish Anti‐Tuberculosis Association, during the conduct of the study.

S. Toppila‐Salmi reports other from ALK‐Abello, grants and other from Sanofi, grants and other from GSK, Orion Pharma, Clario, AstraZeneca, outside the submitted work.

T. Zuberbier reports honoraria for lectures from Amgen, AstraZeneca, AbbVie, ALK ‐Abelló, Almirall, Astellas, Bayer Health Care, Bencard, Berlin Chemie, FAES Farma, HAL Allergie GmbH, Henkel, Kryolan, Leti, L'Oreal, Meda, Menarini, Merck Sharp & Dohme, Novartis, Nuocor, Pfizer, Sanofi, Stallergenes, Takeda, Teva, UCB, and Uriach; Fees for industry consulting were received from Abivax, Almirall, Bluprint, Celldex, Celltrion, Novartis, and Sanofi; in addition he declares non‐paid organisational affiliations: Committee member, ‘Allergic Rhinitis and its Impact on Asthma’ (ARIA), Member of the Board, German Society for Allergy and Clinical Immunology (DGAKI), Head, European Centre for Allergy Research Foundation (ECARF), President, Global Allergy and Asthma Excellence Network (GA^2^LEN), and Member, Committee on Allergy Diagnosis and Molecular Allergology, World Allergy Organisation (WAO).

N. Pham‐Thi reports other from ALK, Stallergenes, outside the submitted work.

L. Taborda‐Barata reports personal fees from LETI, personal fees from Sanofi, personal fees from Diater, outside the submitted work.

D. Larenas Linnemann reports personal fees from ALK, Astrazeneca national and global, Bayer, Chiesi, Grunenthal, Grin, GSK national and global, Viatris, Menarini, MSD, Novartis, Pfizer, Sanofi, Siegfried, Carnot, Syneos Health, grants from Abbvie, Bayer, Lilly, Sanofi, Astrazeneca, Pfizer, Novartis, Pulmonair, GSK, Chiesi, outside the submitted work; and Editor in chief of Immune System (Karger); Member of asthma committee ACAAI; Subgroup chair of allergen immunotherapy Practice parameter update JTF AAAAI/ACAAI 2024; Member of allergen immunotherapy committee AAAAI; Chair of allergen immunotherapy committee CMICA; Member of allergic asthma task force EAACI.

M. Giovannini reports fees from Sanofi, Thermo Fisher Scientific, outside the submitted work.

The other authors have nothing to declare, outside the submitted work.

## Supporting information


**Data S1:** all70055‐sup‐0001‐Supinfo.docx.

## Data Availability

The data that support the findings of this study are available from the corresponding author upon reasonable request.
