# Supplementary material for: Comparison of Allergic Rhinitis Treatments on Patient Satisfaction: A MASK‐air and EAACI Methodological Committee Report
Source: Allergy. 2025 Sep 26;80(12):3319–30. doi: 10.1111/all.70055 (PMC12666759; doi:10.1111/all.70055)
Supplement: Supplementary file 1 — Data S1: all70055‐sup‐0001‐Supinfo.docx. [file ALL-80-3319-s001.docx]

**Supplementary material**

**Supplementary Table 1. Visual analogue scales (VASs) available in MASK-air**

| **VAS** | **Question** |
| --- | --- |
| VAS Global Allergy Symptoms | Overall, how much are your allergic symptoms bothering you today? |
| VAS Nose | How much are your nose symptoms bothering you today? |
| VAS Eyes | How much are your eye symptoms bothering you today? |
| VAS Asthma | How much are your asthma symptoms bothering you today? |
| VAS Satisfaction | How satisfied are you with the treatment you have taken today for rhinitis? |

**Supplementary Table 2. Frequency of MASK-air^®^ users and daily monitoring questionnaire days per country**

| **Country** | ***N* days** | ***N* participants** |
| --- | --- | --- |
| Argentina | 473 (1.7) | 59 (3.5) |
| Australia | 4 (0.01) | 2 (0.1) |
| Austria | 122 (0.4) | 16 (0.9) |
| Belgium | 176 (0.6) | 4 (0.2) |
| Brazil | 587 (2.1) | 46 (2.7) |
| Canada | 1 (<0.01) | 1 (0.1) |
| Czech Republic | 802 (2.8) | 58 (3.4) |
| Denmark | 33 (0.1) | 2 (0.1) |
| Ecuador | 113 (0.4) | 6 (0.4) |
| Finland | 529 (1.9) | 5 (0.3) |
| France | 937 (3.3) | 55 (3.3) |
| Germany | 6296 (22.3) | 269 (15.9) |
| Great Britain | 117 (0.4) | 12 (0.7) |
| Greece | 1661 (5.9) | 142 (8.4) |
| Hungary | 1683 (6) | 94 (5.6) |
| Italy | 1903 (6.8) | 156 (9.2) |
| Japan | 124 (0.4) | 29 (1.7) |
| Lithuania | 1917 (6.8) | 116 (6.9) |
| Mexico | 757 (2.7) | 56 (3.3) |
| Netherlands | 43 (0.2) | 6 (0.4) |
| Poland | 2742 (9.7) | 200 (11.8) |
| Portugal | 4138 (14.7) | 185 (10.9) |
| Slovenia | 1002 (3.6) | 46 (2.7) |
| Spain | 1092 (3.9) | 61 (3.6) |
| Sweden | 5 (0.02) | 3 (0.2) |
| Switzerland | 31 (0.1) | 9 (0.5) |
| Turkey | 830 (2.9) | 45 (2.7) |
| Ukraine | 44 (0.2) | 5 (0.3) |
| United States | 15 (0.1) | 3 (0.2) |

**Supplementary Table 3. Distribution of demographic and clinical variables (A) according to the use of different treatment classes and (B) in relation to the levels of the visual analogue scale (VAS) of rhinitis treatment satisfaction**

| **A. Distribution of demographic and clinical variables according to the use of different treatment classes** | | | | | |
| --- | --- | --- | --- | --- | --- |
|  | **Age – mean (SD)** | **Females – *n* (%)** | **Asthma – *n* (%)** | **Conjunctivitis – *n* (%)** | **Use of medication for asthma – *n* (%)** |
| INAH | 38.8 (13.4) | 33 (56.9) | 17 (20.3) | 30 (51.7) | 9 (15.5) |
| INCS | 44.5 (17.3) | 443 (57.6) | 272 (35.4) | 318 (41.4) | 221 (28.7) |
| INAH+INCS | 42.0 (15.9) | 219 (54.5) | 152 (37.8) | 158 (39.3) | 134 (33.3) |
| OAH | 40.6 (15.0) | 728 (58.2) | 423 (33.8) | 589 (47.1) | 323 (25.8) |
| **B. VAS satisfaction levels according to patients’ demographic and clinical characteristics** | | | | | |
|  | | **INAH** | **INCS** | **INAH+INCS** | **OAH** |
| Age | |  |  |  |  |
| ≤40 years – median (P25-P75) | | 57 (31-77) | 80 (57-92) | 79 (57-91) | 80 (57-92) |
| >40 years – median (P25-P75) | | 91 (76-96) | 86 (72-94) | 78 (30-91) | 84 (60-93) |
| Sex | |  |  |  |  |
| Females – median (P25-P75) | | 71 (46-93) | 83 (66-93) | 84 (66-93) | 83 (63-93) |
| Males – median (P25-P75) | | 78 (56-92) | 85 (67-93) | 66 (16-88) | 81 (51-92) |
| Presence of asthma | |  |  |  |  |
| No asthma – median (P25-P75) | | 83 (52-94) | 84 (63-93) | 83 (55-92) | 84 (60-93) |
| Asthma – median (P25-P75) | | 67 (42-82) | 85 (70-93) | 76 (46-88) | 81 (61-91) |
| Presence of conjunctivitis | |  |  |  |  |
| No conjunctivitis – median (P25-P75) | | 60 (34-75) | 85 (62-93) | 79 (54-91) | 84 (60-93) |
| Conjunctivitis – median (P25-P75) | | 89 (69-95) | 84 (68-92) | 77 (30-91) | 80 (55-90) |
| Use of medication for asthma | |  |  |  |  |
| No use of medication – median (P25-P75) | | 81 (51-93) | 84 (61-93) | 81 (53-92) | 84 (59-93) |
| Use of medication – median (P25-P75) | | 65 (48-79) | 84 (69-92) | 76 (45-89) | 80 (57-90) |

INAH=Intranasal antihistamines; INCS=Intranasal corticosteroids; OAH=Oral antihistamines; P25-P75=Percentile 25-75; SD=Standard-deviation

**Supplementary Figure 1. Directed acyclic graph representing the relationships between (i) medication use, (ii) satisfaction or use of co-medication and (iii) potential confounders**

**
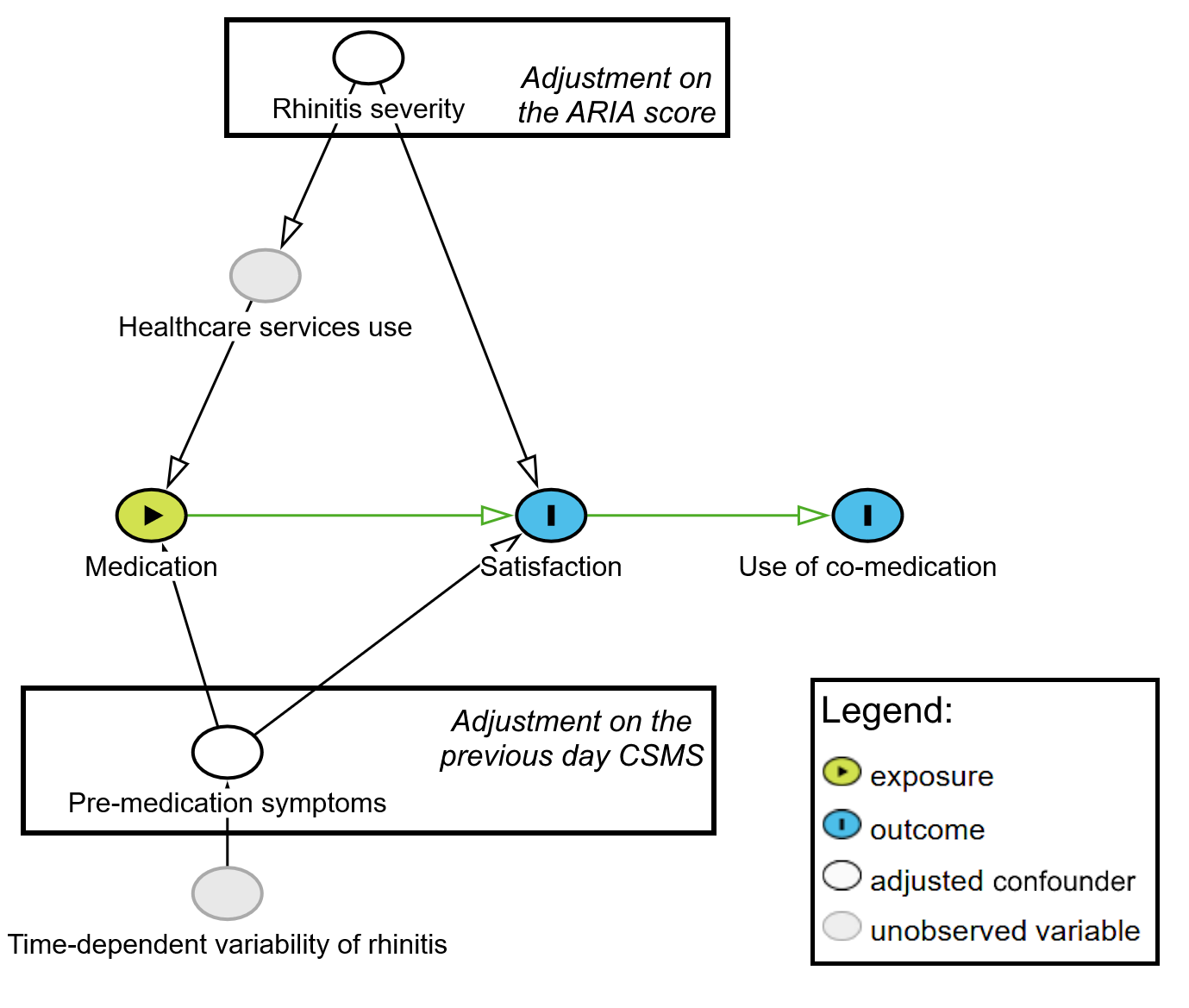
**

ARIA=Allergic Rhinitis and its Impact on Asthma; CSMS=Combined symptom-medication score

In our study, we aimed to assess the association between use of different medications (exposure) and treatment satisfaction levels or frequency of co-medication use (outcomes). However, rhinitis severity and time-dependent variability of rhinitis may be two relevant confounders in regards to this association. In this context, we adjusted for the patient ARIA score (an indicator of rhinitis severity) and for the CSMS of the previous day (as an indicator of pre-medication symptoms).

**Supplementary Figure 2. Flow diagram illustrating participant selection**

**
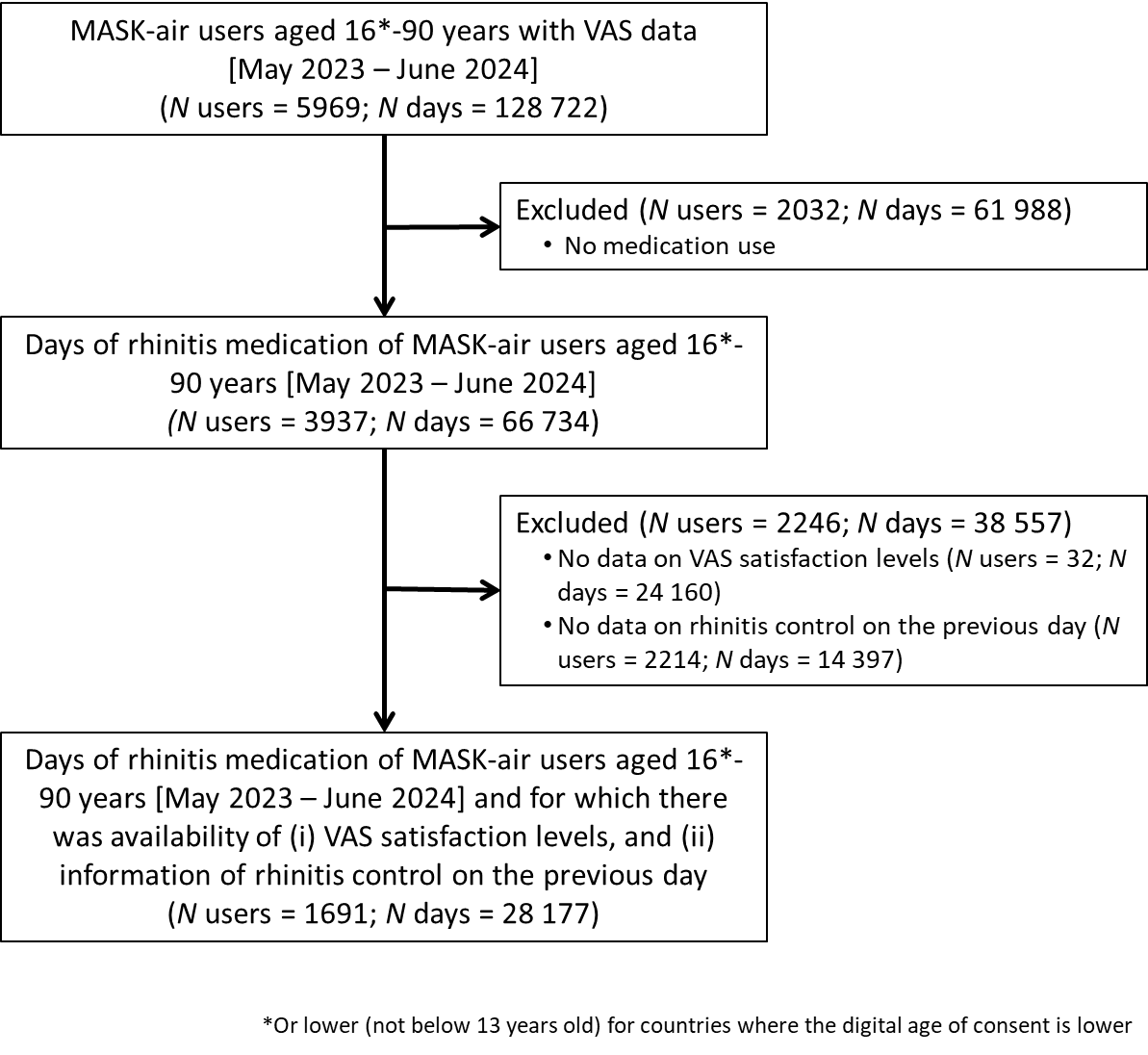
**
